# Potential Therapeutic Effect of Bee Pollen and Metformin Combination on Testosterone and Estradiol Levels, Apoptotic Markers and Total Antioxidant Capacity in A Rat Model of Polycystic Ovary Syndrome

**DOI:** 10.22074/IJFS.2020.134604

**Published:** 2021-03-11

**Authors:** Leila Naseri, Mohammad Rasoul Khazaei, Mozafar Khazaei

**Affiliations:** 1 Student Research Committee, Kermanshah University of Medical Sciences, Kermanshah, Iran; 2 Fertility and Infertility Research Center, Health Technology Institute, Kermanshah University of Medical Sciences, Kermanshah, Iran

**Keywords:** Apoptosis, Bee Pollen, Estradiol, Metformin, Polycystic Ovarian Syndrome

## Abstract

**Background:**

Polycystic ovary syndrome (PCOS) is associated with metabolic disorder as well as infertility. Many traditional remedies have been reported to show estrogenic and antioxidant potential. Bee pollen is a natural com- pound, reported as one such remedy. The present study aimed to investigate the effects of BP extract and metformin (MET) on estradiol (E2) and testosterone (T) levels, apoptotic markers, and total antioxidant capacity (TAC) inarat model of PCOS.

**Materials and Methods:**

In this experimental study, 54 female Wistar (n=6/group) rats received 2 mg of estradiol valerate (EV) intramuscularly and 6 additional rats were considered the control without EV injection. The rats were treated with BP (50, 100, and 200 mg/kg), MET (300 mg/kg) and BP+MET (50 BP+300 MET, 100 BP+300 MET, and 200 BP+300 MET mg/kg). Serum levels of E2 and T were assessed by ELISA method. TAC of serum was also determined. The expressions of *Bcl-2, Bax and Caspase-3 (Cas-3)*, and *Sirt-1* genes were evaluated by real-time poly- merase chain reaction (PCR). Data were statistically analyzed using one-way ANOVA.

**Results:**

In the untreated PCOS group E2 and T levels (P<0.01), and *Bcl-2* (P=0.007) expression were increased, but TAC (P=0.002) and expression of *Bax* (P=0.001), *Cas-3* and *Sirt1* (P<0.01) were decreased significantly. The levels of E2 and T, as well as the expressions of *Bcl-2* were decreased in all treated groups compared to the untreated PCOS group (P<0.01). On the other hand, TAC and expression of *Bax, Cas-3* and *Sirt1* were increased in the BP- and MET-treated groups (P<0.05).

**Conclusion:**

BP and MET synergistically improved serum E2, T and TAC levels, and expression of apoptotic genes.

## Introduction

Polycystic ovary syndrome (PCOS) as one of the most prevalent disorders of the endocrine system is among the leading causes of non-ovulatory infertility. It is also the main common endocrine, metabolic, and genetic disorder characterized by an ovulation, polycystic ovary, and biochemical and clinical signs of hyper androgenism (1). PCOS is associated with adrenal-pituitary-hypothalamic dysregulations and is observed in about 4 to 18% of women of fertility age (2).

PCOS women have higher levels of luteinizing hormone (LH), testosterone (T), cholesterol, and triglycerides but in contrast, they have lower levels of follicle stimulating hormone (FSH), sex hormone-binding globulin (SHBG), and high-density lipoproteins (HDLs) (2). The most common complications of PCOS are non-ovulation, type 2 diabetes, cardiovascular diseases, and menstrual disorders, which can lead to infertility if remain untreated. In PCOS women, the functions of both insulin and its receptor are normal; however the signaling cascade activated following the binding of insulin to its receptor, is impaired (3). Furthermore, apoptosis of granulosa cells, which is an essential process for the normal development of follicles, has been highlighted in the pathogenesis of PCOS (4).

PCOS treatment includes medications such as medroxyprogesterone, cyproterone acetate, spironolactone, metformin (MET), and birth control pills. MET as one of the drugs used in diabetes, has recently been applied to treat PCOS, as it improves menstruation and ovulation patterns within 2 to 3 months (5). In addition to chemical drugs, many herbal/traditional remedies are now used to relieve PCOS symptoms. Herbal and natural compounds have wide popularity because of their lower side effects, and greater compatibility with the body, as well as their extensive variety.

Bee pollen (BP) is a circular seed-like particle (2.5-250 μm diameter) covered by a protective wall and contains significant amounts of proteins, lecithin, active enzymes, folic acid, vitamins, especially B family, and other minerals. Bees combine small volumes of their saliva with pollens accumulated from plants, to produce bee bread. The product of this process is stored in corbiculae on the dorsal tibia. Based on plant species, pollens show variable morphological shapes. Furthermore, their color is variable from light yellow to black. Regarding the size, pollens may be seen as grains of ten to hundreds of micrograms. The beneficial effects of this compound on prostate inflammation, cancer, chronic alcoholic disease, burn wounds, and depression have been recognized (6). 

Various studies have shown that BP has many valuable effects in improving infertility, allergies, and obesity (7-9). BP is a potential source of natural antioxidants including vanillic, protocatechuic acid, gallic, and p-Coumaric acid as well as flavonoids such as hesperidin, rutin, kaempferol, apigenin, luteolin, quercetin, and isorametin (10). These compounds have shown antioxidant activity by inactivating and removing free radicals and reactive oxygen species (ROS such as H_2_ O_2,_ O_2_ •- and •OH). Accordingly, Šarić et al. (11) attributed the potent antioxidant effects of BP to its phenolic compounds such as quercetin, isorhamnetin, galangin, chrysin, and pinocembrin. In another study, Sousa et al. (12) stated that anti-oxidative components of BP, especially cyanidine and kaempferol, inhibited CYPs, and protected Caco-2 enterocyte intestinal cells against tert-Butyl hydroperoxide (t-BHP)- induced oxidative stress. Given the aforementioned and unique properties of BP, and the fact that the effects of BP on PCOS have not been investigated yet, the aim of the present study was to assess the effects of BP alone and in combination with MET on sex hormone levels, total antioxidant capacity (TAC), and expression of anti- and pro-apoptotic genes, in a rat model of PCOS.

## Materials and Methods

### Animals

In this animal experimental study, 54 mature female Wistar rats (180-210 g) were maintained under standard laboratory conditions (22-24°C and12-hour dark-light cycle) and they had free access to food and water ad libitum. Rats were selected based on two to three regular estrous periods during 8-10 days of vaginal smear evaluation (13). BP was collected from beehives in East Azerbaijan province, Iran. It was dissolved in distilled water (DW) (5 g in 20 ml) by stirring for 2 hours. After filtration, the suspension was centrifuged at 10,000 g for 20 minutes and the supernatant was kept in the freezer until use. Before analysis, the suspension was thawed and dissolved in DW. Body weight was measured at the binging and the end of the experimental period. Also, all experimental procedures in this study were reviewed and approved with the Guidelines of the Ethical Committee for Research on Laboratory Animals at Kermanshah University and Medical Sciences (IR.KUMS.REC.1398.496). 

### Polycystic ovary syndrome induction

PCOS was induced by a single intramuscular injection of estradiol valerate (EV, 2 mg dissolved in 0.2 ml olive oil) (Aburaihan, Iran). During the 60 days of the study, vaginal smears were obtained to determine abnormal estrous cycle and persistent vaginal cornification (PVC) and irregularity of the estrous cycle (14). Furthermore, to establish a PCOS diagnosis, 6 rats were selected randomly and the levels of E2 and T were measured. 

### Treatment groups

The rats were divided into 9 groups (n=6/group) as follows: 1: Control, 2: PCOS (without intervention), 3-5: PCOS with BP at the doses of 50, 100 or 200 mg/kg, 6: PCOS with MET (300 mg/kg) (15), and 7-9: PCOS with MET 300 (mg/kg)+50, 100, or 200 mg/kg BP. All the treatments were performed for 21 days by gavage (16). 

### Serum analysis

At the end of the study, rats were sacrificed under general anesthesia induced by 100 mg/kg ketamine and 10 mg/kg xylazine (Alfasan, Warden-Holland) (intraperitoneally) (17). The blood samples were taken from the hearts and the serum was isolated (3000 rpm for 15 minutes) and stored at -20°C. Then, the levels of E2 and T were measured using rat ELISA Kits (ZellBio, Germany, Histogenotech, testosterone: ZB-10259C, estradiol: ZB10176C). TAC of serum was measured by the ferric reducing ability of plasma (FRAP) method as explained in our previous studies (18).

### Quantitative real-time polymerase chain reaction

The right ovaries were removed and stored in liquid nitrogen for gene expression analysis. The ovarian tissue (30 mg) was used to extract total RNA by a kit protocol (BioFact kit, South Korea, Noavaranteb, PR101-050/ PR101-100). The quality of the RNA was checked by NanoDrop spectrophotometers 2000c (Thermo Science, USA) and determining the absorbance ratios of A260/ A280 and A260/A230 nm. The DNA was synthesized using the BioFact kit (BioFact RT Series, South Korea, Noavaranteb, BR63110-96). The reaction mixture was prepared in a net volume of 20 μL. The polymerase chain reaction (PCR) master mix of SYBR Green I (TaKaRa, South Korea, Noavaranteb, DQ385-40H) was added to the reactions tube (10 μL). Other constituents of the reaction mixture included forward (1 μL) and reverse (1 μL) primers (400 nM), cDNA (1 μL), and ddH2O (7 μL). The primers were designed using Gene Runner and Primer Express software v.3.0 (Applied Biosystems, Foster City, USA) and blasted in NCBI (Table 1). Realtime PCR thermal program consisted of an initial incubation at 70°C for 45 minutes. Then, 38 to 42 cycles were considered [95°C for 30 seconds 60°C (annealing and extension) for 1 minutes]. Melting curve analysis was carried out between 60 and 95°C (1°C increments) for 5 s at each step.

**Table 1:** Primers for real-time polymerase chain reaction


Gene	Primer sequence (5ˊ-3ˊ)	Tm (°C)

*Bax*	F: GCTACAGGGTTTCATCCA R: ACATCAGCAATCATCCTCT	52.8 52.5
*Bcl-2*	F: ATCGCTCTGTGGATGACT R: CAGCCAGGAGAAATCAAACA	55 54.3
*Caspase-3*	F: GTGGAACTGACGATGATATGG R: GCAAAGTGACTGGATGAACC	56.1 55.3
*Sirt-1*	F: AAGACCAGTAGCACTAATTCCAAGT R: GCCACCTAACCTATGACACAACT	59.3 59
*β-Actin* (Internal reference gene)	F: CTCATAGATGGGCACAGTGTGGG R: TGACCCAGATCATGTTTGAGACC	61.9 59.3


The High ROX BioFact™ 2X Real-Time PCR Smart mix SYBR Green PCR master mix was applied to determine the expression of target genes (*Caspase-3, Bax, Bcl2* and *Sirt-1*). The reactions were carried out in a device manufactured by the Applied Biosystems (StepOne™ Real-Time PCR System, USA). The experiments were carried out in duplicate. The housekeeping gene, *β-Actin*, was used as the internal control. Gene expression levels were measured by using below formulas and finally by Ct (2^-ΔΔt^) (fold changes) method.

∆∆CT=[(mCTtarget-mCTreference) test sample- (mCTtarget-mCTreference) control sample]

And finally: Expression level of target gene=2^-ΔΔct ^

### Statistical analysis 

Considering the normal distribution of the data confirmed by the Kolmogorov-Smirnov test (P>0.05), differences among data were statistically analyzed using one-way ANOVA followed by Tukey’s test (a P<0.05 was considered significant). Data analysis was done by SPSS-16 software (SPSS, Inc., Chicago, IL, USA) and data are presented as mean ± SD. The GraphPad Prism software package version 8 (Graph Pad Prism Software Inc., San Diego, California) was used to draw data charts.

## Results

**Fig.1:**
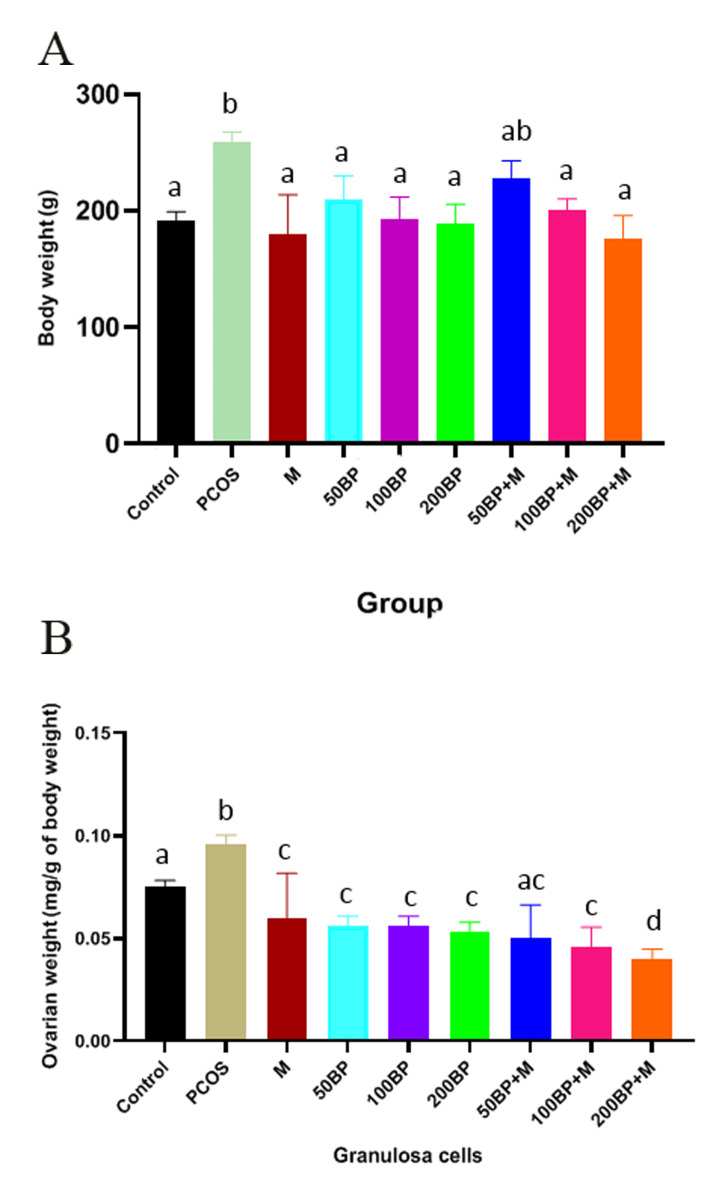
Effect of BP and MET on body and ovarian weight in different groups. **A.** Body weight and **B.** Ovarian weight. Different letters indicate significant differences between groups and the same letters indicate no significant differences between groups. BP; Bee pollen, M; Metformin, and PCOS; Polycystic ovary syndrome.

The body (P=0.002) and ovary (P<0.001) weight increased in the PCOS group. Body weight significantly decreased in groups treated with 200 mg/kg BP (P=0.02), and combination of Met and BP 100 (P=0.03) and 200 mg/kg (P=0.06) compared to the PCOS group (Fig.1A). The body weight was significantly lower in all groups treated with (50, 100 and 200 mg/kg BP (P<0.001), and combination 50 (P=0.003), 100 and 200 mg/kg BP+MET (P<0.001) and MET (P=0.04), Fig.1B).

### Sex hormones

**Fig.2:**
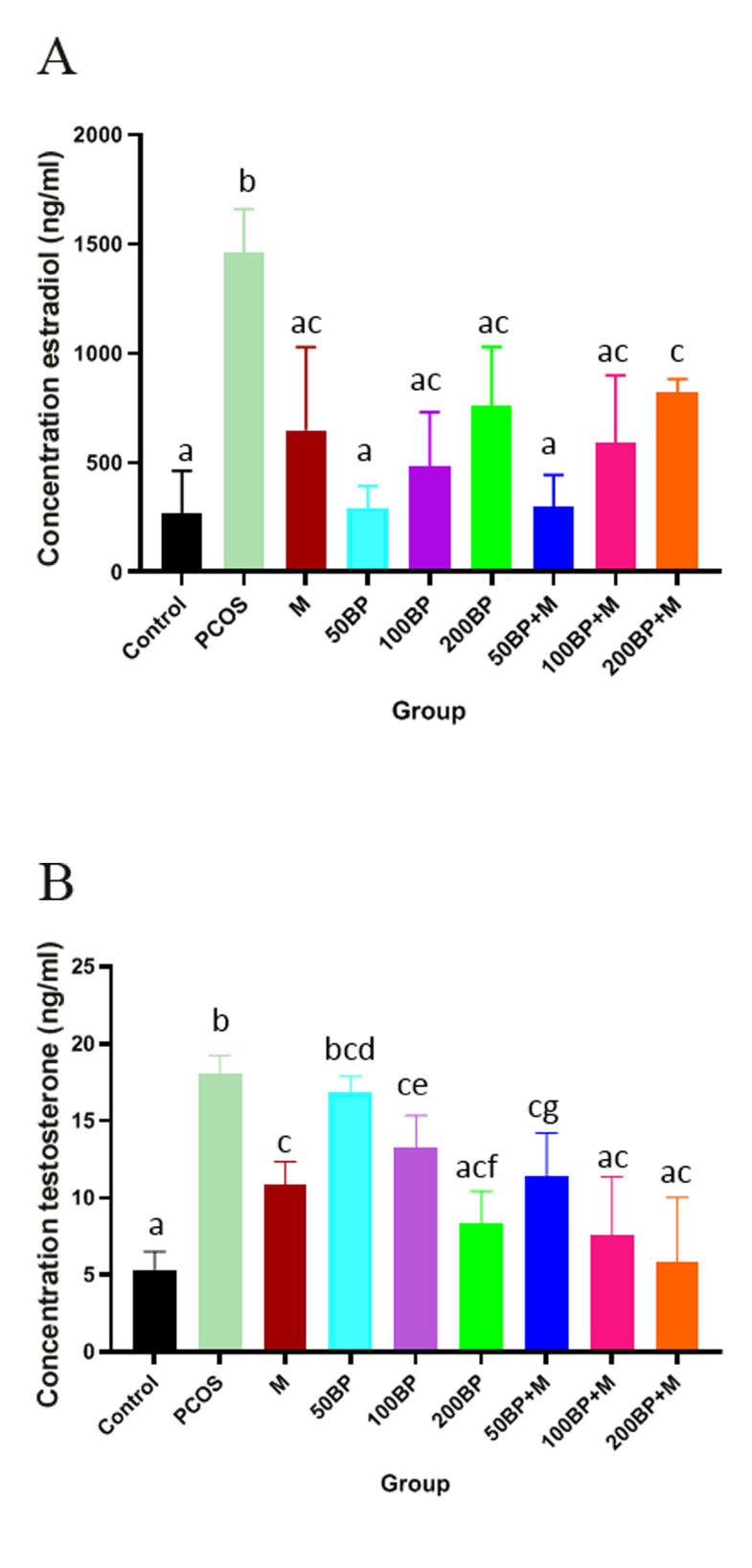
Comparison of Serum levels of sex hormones in the different experimental groups. **A.** Serum levels of E2 in the experimental groups: PCOS (P<0.01), 50 mg/kg (P=0.01), 100 mg/kg (P=0.005) and 200 mg/kg (P=0.001) of BP, 50 mg/kg BP+M (P=0.005), 100 mg/kg BP+M (P=0.003) and 200 mg/kg BP+M (P=0.001). Different letters indicate significant differences between groups and the same letters indicate no significant differences between groups. **B.** Serum testosterone in the experimental groups (mean ± SD). PCOS (P<0.01), 50 mg/kg (P=0.3), 100 mg/kg (P=0.006) and 200 mg/kg (P=0.001) of BP, METtreated (P<0.01), combination of BP and MET, testosterone level significantly decreased comparing to PCOS (P<0.01). Different letters indicate significant differences between groups and the same letters indicate no significant differences between groups. BP; Bee pollen, M; Metformin (MET), and PCOS; Polycystic ovary syndrome.

PCOS induction by EV significantly increased the serum levels of E2 and T while their levels in the treatment groups reduced (P<0.01). The E2 levels were significantly lower in all groups treated with 50 (P=0.01), 100 (P=0.005) and 200 (P=0.001) mg/kg BP, and combination of 50 (P=0.005), 100 (P=0.003) and 200 (P=0.001) mg/ kg of BP with MET compared to the PCOS group. E2 reached the control level in the group treated with 50 mg/ kg BP+MET. Although, the group treated with 200 mg/kg BP+MET showed a significant increase compared to the control group (P=0.007, Fig.2A). 

While T level significantly increased in the PCOS group, it decreased by BP alone or in combination with MET dose-dependently (P<0.05). MET treatment reduced T level (P<0.01), but it did not reach the control level. The group treated with 50 mg/kg BP showed no significant difference compared to the PCOS group (P=0.3), but the group treated with 100 (P=0.006) and 200 mg/kg (P=0.001) of BP had significant differences (Fig.1B). Furthermore, in rats treated with a combination of BP and MET, T level significantly decreased (P=0.001) and it reached the control level in 200 mg/kg+MET group (Fig.2B). 

### Total antioxidant capacity

**Fig.3:**
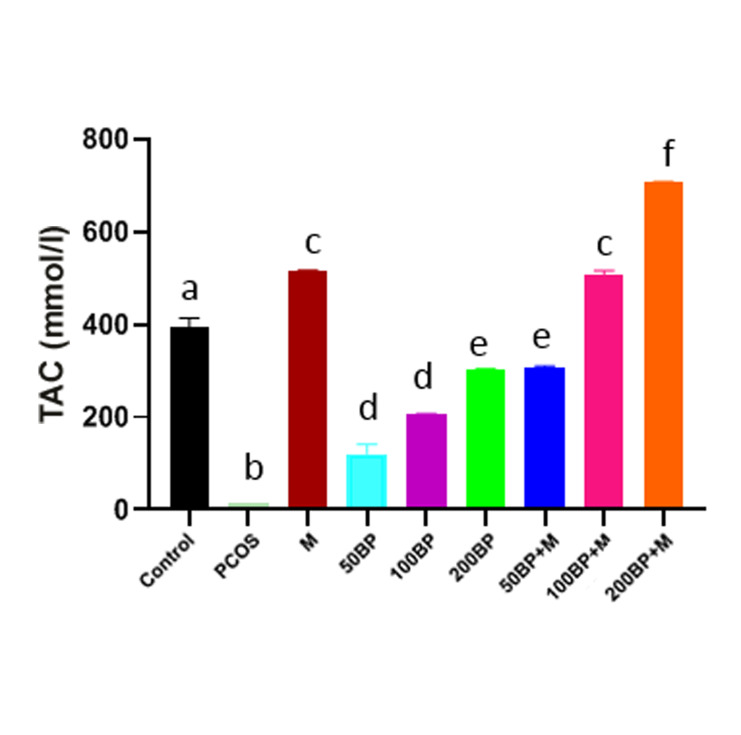
Comparison of TAC between the study groups. In all treatment groups, TAC significantly and dose-dependently increased compared to PCOS group (P<0.01). 100 mg/kg; (P=0.03), and 200 mg/kg; (P=0.006) and MET group (P=0.03). Different letters indicate significant differences between groups and the same letters indicate no significant differences between groups. BP; Bee pollen, M; Metformin (MET), and PCOS; Polycystic ovary syndrome.

The TAC severely decreased in the PCOS group (P=0.002). In all treated PCOS groups with BP or BP+MET, it significantly increased in a dose-dependent manner (P<0.01). Its elevation did not reach the control levels in the group treated with BP alone, but it was higher than control in rats treated with combinations of MET and BP [(100 mg/kg (P=0.03), and 200 mg/kg (P=0.006) groups]. TAC level also significantly increased in rats treated with MET (P=0.03) (Fig.3).

### Genes expressions

**Fig.4:**
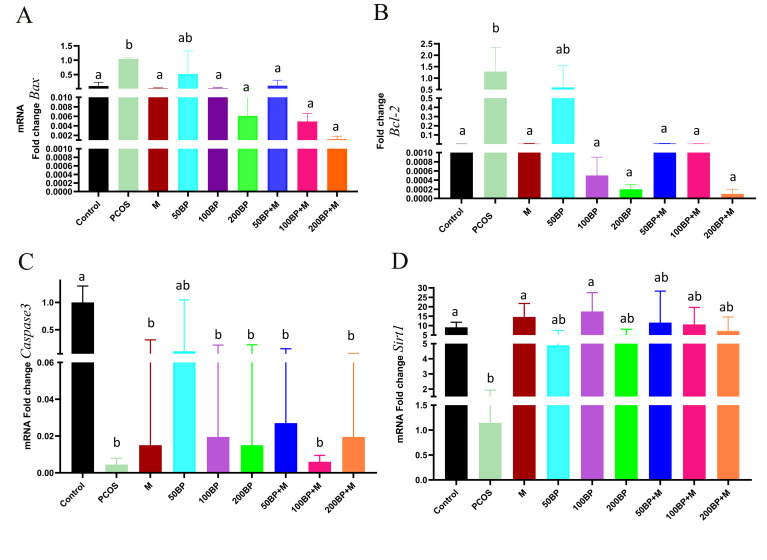
mRNA expressions of *Bax*, *Bcl-2*, Caspase-3 and *Sirt-1* genes in rat ovarian tissue in study groups. **A.** *Bax* (P<0.001), **B. ***Bcl-2*, **C.** *Caspase-3* (P<0.01) and **D.** *Sirt-1* (P<0.001). Different letters indicate significant differences between groups and the same letters indicate no significant differences between groups. BP; Bee pollen, M; Metformin (MET), and PCOS; Polycystic ovary syndrome.

The expression of Bax gene was significantly reduced in the PCOS group (P=0.001), it expression significantly increased in the both BP-treated groups (P<0.001). The Bax expression in the groups treated with 50 mg/kg BP+MET (P=0.002), 100 mg/kg BP+MET, 200 mg/kg BP+MET, and MET alone was increased significantly compared to the PCOS group (P<0.001, Fig.4A). The expression of *Bcl-2* gene was significantly elevated in the PCOS group (P=0.007), it significantly decreased in the groups treated with 100 mg/kg BP (P=0.007), 200 mg/kg BP (P=0.007), MET (P<0.01), and combination of MET and BP (50,100 and 200 mg/kg, P<0.01) compared to the PCOS group (Fig.4B).

*Caspase-3* gene expression significantly decreased in the PCOS group (P<0.01). However, the expression of this gene increased in treated groups (P=0.09, Fig.4C). The expression of *Sirt-1* gene was significantly reduced in the PCOS group (P=0.002). In all PCOS groups treated with BP or BP+MET, the expression of this gene increased but this was not significant except for the 100 mg/kg BE (P=0.02) and MET (P=0.04, Fig.4D).

## Discussion

BP alone and in combination with MET significantly reduced the levels of T, body weight and *Bcl-2* gene expressions, and increased E2 and TAC significantly, as well as expressions of *Bax*, *Caspase-3*, and *Sirt-1* genes in the PCOS treated group. In this study, higher BP doses were more effective. Changes in the levels of *Bax*, TAC, and T were significant for 100 and 200 mg/kg BP doses. Examining apoptotic markers and sex hormones indicated the beneficial effects of the 200 mg/kg BP.

Women with PCOS have abnormal androgen and E2 production, which depends on the extent of LH stimulation. BP exerts E2-like activity due to the presence of phytosterols such as P- and β-sitosterol (19). These are cholesterol-like compounds that can interfere with cholesterol uptake and therefore, reduce synthesis of androgenic hormones, especially T (20). Steroid hormones produced by ovaries act as autocrine factors and play an important role in controlling ovarian cell death. E2 acts as an key factor for the survival of both granulosa cells and corpus luteum. Progesterone also maintains the survival of granulosa cells. Although the mechanism of action of steroid hormones is not completely understood, they can partly act through inducing the production of BCL-2 (21). Increased anti-apoptotic factors lead to the preservation of ovarian cysts and the progression of PCOS (22). In this syndrome, high levels of estrogen prevent apoptosis, which is a feature of PCOS. In the intervention groups, BP treatment reduced E2 levels and the expressions of antiapoptotic genes, while increased the expressions of proapoptotic genes, which subsequently can induce apoptosis in ovarian cysts and mitigate PCOS manifestations. 

A mutation in the aromatase P450 enzyme can lead to ovarian dysfunction and increased androgens’ levels as a trigger factor for PCOS. The aromatase enzyme catalyzes E2 biosynthesis from androgens (23). β-sitosterol inhibits the activity of cytochrome P450 in the microsomes of human liver, inhibiting the conversion of cholesterol to pregnenolone and other steroid hormones (24). Malini and Vanithakumari (25) showed that β-sitosterol reduced the production of cholesterol and gonadal steroids. Consistent with these studies, our results also suggested that BP can reduce production of steroid hormones probably by β-sitosterol. Body weight might be one of the main clinical features of PCOS patients (26). In this study, it was observed that BP and MET reduce the weight of rats and ovaries.

BP is a phenolic-rich compounds with anti-androgenic properties (27) and suppresses the binding of dehydrotestosterone to its receptor, leading to reduced T secretion. Flavonols are structurally similar to E2, so it is hypothesized that these compounds may compete with androgens for binding to receptors, affecting internal androgen levels. Other flavonols are apigenin and kaemferol; apigenin is an anti-androgenic compound with inhibitory effects on cytochrome P450 activity (28). It reduced T secretion in the adrenal cortex (29). Apigenin is also present in BP which can explain anti-estrogenic effects (30) observed in the present study.

In the study of Wang et al. (31), apigenin competitively inhibited the binding of flunitrazepamto the GABA receptor and reduced the LH secretion. Furthermore, researchers have shown that kaempferol can promote E2-like activities (27). It can inhibit the binding of E2 to alpha-fetoprotein (AFP), a serum estradiol-binding protein. The interaction of biological flavonoids with AFP can limit the availability of E2 for binding to target cells (32). In the present study, inhibition of E2 binding to AFP by BP ingredients, can explain E2 reduction in PCOS rats. Monsefi et al. (33) also showed that kaempferol available in Anethum graveolens reduced E2 level through binding to AFP.

BP also significantly increased TAC in the treatment groups. Murri et al. (34) reported abnormal levels of circulating markers of oxidative stress in women with PCOS proposing a role for these markers in PCOS pathophysiology. Oxidative stress is an imbalance between ROS production and antioxidant system activity. A variety of female reproductive functions can be disturbed by the effect of oxidative mediators. The most important of such results is abnormal maturation of oocytes, as well as disturbances in ovulation (35). The normal growth of the inner theca layer is essential for ovarian function. Free radicals and oxidative mediators can disrupt regular growth and induce apoptosis in this layer. In PCOS women, a direct relationship has been shown between decreased oxidative stress and increased oocyte maturation (36). So, antioxidants can help improving PCOS symptoms (10).

We also investigated the effect of BP on the gene expressions of anti-apoptotic (*Bcl-2*) and proapoptotic (*Bax* and *Caspase-3*) markers. To our knowledge, no studies have been performed to date on the effects of BP on apoptosis of ovarian cells in PCOS. In this study, while anti-apoptotic genes were reduced, pro-apoptotic genes were increased in the PCOS group which was consistent with previous studies. Das et al. (37) reported that the expressions of pro-apoptotic genes: including *Bax* were significantly reduced in PCOS women. On the other side, the expression of *Bcl-2* was significantly elevated in these patients indicating a low rate of apoptosis in their ovarian cysts. Likewise, Isobe and Yoshimura (38) showed that the expressions of caspase-3 and BAX was significantly reduced in bovine cystic ovarian disease.

In another report, Salvetti et al. (22) investigated the proliferation and apoptosis rates, as well as the levels of some proteins (i.e. BAX, BCL-xL, BCL-w, and BCL-2) involved in these processes in different types of ovarian follicles in PCOS rats. They asserted that the reduced apoptosis in follicles of these rats can be involved in the formation and persistence of ovarian cysts. These findings indicate the roles of BCL-2 and BAX in apoptosis of ovarian granulosa cells in PCOS. In parallel, we also observed decreased levels of apoptotic markers in ovaries in a rat model of PCOS induced by injection of EV.

During the development of the female reproductive system, apoptotic cell death shares a substantial role in the normal formation of ovarian follicles. Follicular granulosa cell apoptosis occurs at childbearing age. These cells are the same cells affected in women with PCOS. BCL-2 inhibits apoptosis through suppressing the release of caspase-9 by reducing the permeability of the mitochondrial membrane. On the other hand, the pro-apoptotic BAX counteracts the function of BCL-2 by facilitating the release of cytochrome c and other apoptotic mediators from mitochondria through increasing the permeability of its outer membrane. The balance of antiand pro-apoptotic mediators is the main regulator of apoptosis in granulosa cells during ovary development and atresia through suppressing caspase-3 activity. According to studies, targeting apoptotic pathways may be a new approach for treating PCOS. In the present study, BP significantly increased apoptosis in granulosa cells. Our results showed that *Sirt-1* expression significantly increased in the BP-treated groups. *Sirt-1* is an NADdependent histone deacetylase, which can improve insulin sensitivity and insulin signal transduction pathways, in insulin-sensitive organs and tissues. Decreased *Sirt-1* expression may be related to the pathogenesis of diseases associated with insulin resistance (39). 

Although the underlying causes of the disease remain unknown, it is believed that insulin resistance plays a key role in the development of PCOS. Recent studies have shown that insulin receptors in skeletal muscles and skin fibroblasts are serine phosphorylated in at least 50% of women with PCOS. Serine phosphorylation regulates the activity of P450. Its aberrant expression triggers insulin resistance and induces elevation of Androgens. This phenomenon, in turn, increases insulin level and Androgen production within ovaries causing premature ovarian atresia and impair ovulation. MET is a drug used to treat non-insulin-dependent diabetes mellitus. This drug decreases insulin and LH levels by suppressing hepatic gluconeogenesis and inducing peripheral glucose uptake (40).

## Conclusion

BP exerts positive effects either individually or synergistically with MET, on E2, T, TAC of serum, and expression of apoptotic genes. BP increased apoptosis in ovarian cysts due to its phytoestrogenic properties. Higher BP doses were more advantageous in this study and producedmore significant changes. Based on the presence of various antioxidants, phytoestrogens, and phenolic compounds, BP could be used as a potential therapeutic agent to treat PCOS.
